# Transcriptome Sequencing and *De Novo* Analysis for Ma Bamboo (*Dendrocalamus latiflorus* Munro) Using the Illumina Platform

**DOI:** 10.1371/journal.pone.0046766

**Published:** 2012-10-03

**Authors:** Mingying Liu, Guirong Qiao, Jing Jiang, Huiqin Yang, Lihua Xie, Jinzhong Xie, Renying Zhuo

**Affiliations:** 1 State Key Laboratory of Tree Genetics and Breeding, Chinese Academy of Forestry, Beijing, People’s Republic of China; 2 Research Institute of Subtropical Forestry, Chinese Academy of Forestry, Fuyang, People’s Republic of China; 3 College of Life Science and Technology, Xinjiang University, Urumqi, People’s Republic of China; Belgian Nuclear Research Centre SCK/CEN, Belgium

## Abstract

**Background:**

Bamboo occupies an important phylogenetic node in the grass family with remarkable sizes, woodiness and a striking life history. However, limited genetic research has focused on bamboo partially because of the lack of genomic resources. The advent of high-throughput sequencing technologies enables generation of genomic resources in a short time and at a minimal cost, and therefore provides a turning point for bamboo research. In the present study, we performed *de novo* transcriptome sequencing for the first time to produce a comprehensive dataset for the Ma bamboo (*Dendrocalamus latiflorus* Munro).

**Results:**

The Ma bamboo transcriptome was sequenced using the Illumina paired-end sequencing technology. We produced 15,138,726 reads and assembled them into 103,354 scaffolds. A total of 68,229 unigenes were identified, among which 46,087 were annotated in the NCBI non-redundant protein database and 28,165 were annotated in the Swiss-Prot database. Of these annotated unigenes, 11,921 and 10,147 unigenes were assigned to gene ontology categories and clusters of orthologous groups, respectively. We could map 45,649 unigenes onto 292 pathways using the Kyoto Encyclopedia of Genes and Genomes Pathway database. The annotated unigenes were compared against Moso bamboo, rice and millet. Unigenes that did not match any of those three sequence datasets are considered to be Ma bamboo unique. We predicted 105 unigenes encoding eight key enzymes involved in lignin biosynthesis. In addition, 621 simple sequence repeats (SSRs) were detected.

**Conclusion:**

Our data provide the most comprehensive transcriptomic resource currently available for *D. latiflorus* Munro. Candidate genes potentially involved in growth and development were identified, and those predicted to be unique to Ma bamboo are expected to give a better insight on Ma bamboo gene diversity. Numerous SSRs characterized contributed to marker development. These data constitute a new valuable resource for genomic studies on *D. latiflorus* Munro and, more generally, bamboo.

## Introduction

Bamboo, the biggest grass, a perennial lignified plant that belongs to Bambusoideae, has considerable economic and cultural significance. Unlike the majority of ∼10,000 grass species that are herbaceous and occupy open habitats such as grassland, bamboo represents the only major lineage of grasses that lives exclusively in forests and grows large woody culms up to 30 cm in diameter and 12 m in height [1]. It is also one of the most important forest resources because of its rapid growth rate, excellent specific strength, and easy machinability. A large number of bamboo species reach their maximum height of 15–30 m in 2–4 months and reach full maturity in about 3–8 years [2]. In addition to remarkable sizes and woodiness, bamboo has rather striking life history characterized by a prolonged vegetative phase lasting up to more than 100 years before flowering. With these unique features, bamboos are important components of tropical and subtropical forest ecosystems, especially in Asia, where they have had a long history of being utilized as garden ornamentals and forest products for making construction material, paper pulp, and furniture. With the realization that bamboo produces high-quality fibers and can be harvested repeatedly without severe destruction of the ecosystems, it becomes an increasingly valuable forest product that could replace a substantial portion of tree-based timber and paper pulp plantation. This highlights another important economic value of grasses in addition to food and renewable energy.

Approximately 1500 commercial applications of bamboo have been identified [3]. In terms of economic and environmental impacts, *Bambusa* and *Dendrocalamus* are the two most important pachymorph rhizome genera [4]. *Dendrocalamus latiflorus* Munro is an evergreen species locally known as ‘tropical giant bamboo’, which forms abundant forests in southern China and southeast Asia and is a valuable natural resource used as building material or for human consumption [5].

The majority of large subfamilies of grasses have already had a great deal of genomic or expressional data available primarily because they possess crop species [6]. However, the subfamily of bamboos, Bambusoideae, which contains more than 1,000 species, have little data available in DNA or protein sequence databases [7], [8]. More serious than the missing link for comparative analyses, this hampers biological investigations of this group of morphologically and physiologically unique and ecologically and economically important grasses.

Sequencing of large genome remains expensive even using next-generation sequencing technologies. Because of the deep coverage and single base-pair resolution provided by next-generation sequencing instruments, RNA sequencing represents an attractive alternative to whole-genome sequencing because it only analyzes transcribed portions of the genome, while avoiding non-coding and repetitive sequences that can make up much of the genome [9]–[12]. The transcriptome is the complete set and quantity of transcripts in a cell at a specific developmental stage or under a physiological condition. The transcriptome provides information on gene expression, gene regulation, and amino acid content of proteins. Therefore, transcriptome analysis is essential to interpret the functional elements of the genome and reveal the molecular constituents of cells and tissues [13], [14]. Although a normalized cDNA library was constructed from young leaves of Ma bamboo and 9,574 high-quality ESTs were generated [15], a comprehensive description of its transcriptome remains unavailable. The increased throughput of next-generation sequencing technologies has shown great potential for expanding sequence databases of not only model species [16]–[20] but also non-model organisms [21]–[26].

In the present study, we performed *de novo* transcriptome sequencing for *D. latiflorus* Munro using the Illumina GA IIx sequencing platform. A total of 103,354 different transcripts and 68,229 unigenes were identified. Also, a large number of simple sequence repeats (SSRs) were determined. To our knowledge, this is the first report on the characterization of the complete transcriptome of *D. latiflorus* Munro. We believe that this new dataset will be a useful resource for future genetic and genomic studies on this species.

## Results and Discussion

### Sequence Analysis and Assembly

To obtain a global overview of the *D. latiflorus* Munro transcriptome and gene activity at nucleotide resolution, a mixed cDNA sample representing diverse developmental stages and tissues of *D. latiflorus* Munro was prepared and sequenced using the Illumina Genome Analyzer. Each sequenced sample yielded 2×72-bp independent reads from either end of a cDNA fragment. After stringent quality assessment and data filtering, 15,138,726 reads (∼2.2 G) with 94.67% Q20 bases (those with a base quality greater than 20) were selected as high quality reads for further analysis. An overview of the sequencing is presented in Table 1. The high quality reads produced in this study have been deposited in the NCBI SRA database (accession number: SRA055083).

**Table 1:** Summary of Illumina transcriptome sequencing for *D. latiflorus* Munro.

Sample	Read Length	No. of Reads	Data	GC (%)	Q20 (%)
Ma bamboo	72+72	15,138,726	2,179,976,544	49.48	94.67

**Figure 1:**
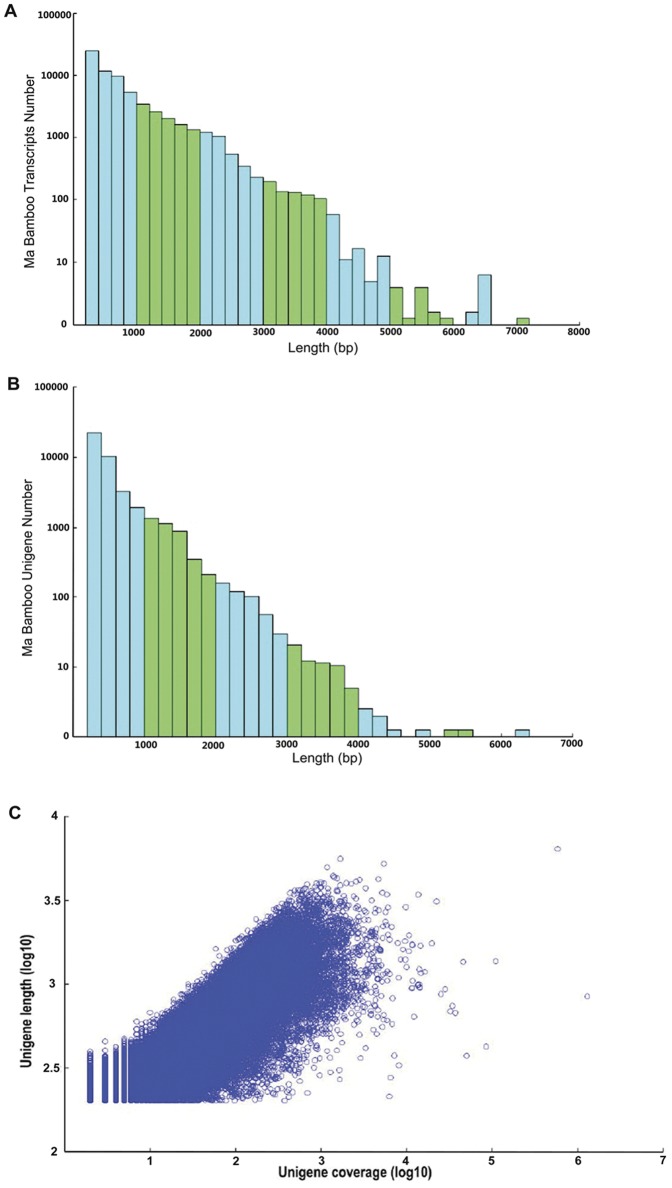
Overview of the *Dendrocalamus latifloru*s Munro transcriptome sequencing and assembly. (A) Length distribution of *D. latifloru*s Munro transcripts. (B) Size distribution of *D. latifloru*s Munro unigenes. (C) Log-log plot showing the dependence of unigene lengths on the number of reads assembled into each unigene.

Using the Trinity *de novo* assembly program, next-generation short-read sequences were assembled into 103,354 scaffolds, with N50 length of 1,132 bp and with mean length of 736 bp. The distribution of scaffolds is shown in Fig 1A. In total, there were 19,236 scaffolds coding for transcripts longer than 1 kb and 5,897 scaffolds coding for transcripts longer than 2 kb. The scaffolds were subjected to cluster and assembly analyses. A total of 68,229 unigenes were obtained, among which 6,375 genes (9.34%) were greater than 1kb. The length distributions of unigenes are shown in Fig 1B, revealing that more than 20,000 unigenes (∼29.3%) are greater than 500 bp. An overview of the assembled scaffolds and unigenes is presented in Table 2. These results demonstrated the effectiveness of Illumina pyrosequencing in rapidly capturing a large portion of the transcriptome. As expected for a randomly fragmented transcriptome, there was a positive relationship between the length of a given unigene and the number of reads assembled into it (Fig 1C). To facilitate the access and utilization of the bamboo transcriptome sequencing data, we have uploaded all the data including the unigene sequences, annotations and relatively highly expressed genes to the ftp site (ftp.biomarker.com.cn) and the category is/zhuory/Munro_Transcriptome/Moso_Bamboo_cDNA (Please contact R. Zhuo for ftp access).

**Table 2:** Summary of Illumina transcriptome assembly for *D. latiflorus* Munro.

	Scaffolds (unique genes)	
Scaffold Length	Total length (bp)	Percentage
0–300	30,162 (28,656)	29.18% (42.00%)
300–500	23,909 (19,546)	23.13% (28.65%)
500–1000	24,158 (13,652)	23.37% (20.01%)
1000–2000	19,236 (5,590)	18.61% (8.19%)
2000+	5,879 (785)	5.69% (1.15%)
Total Length	76,066,622 (33,216,845)	
Count	103,354 (68,229)	
N50_Length	1,132 (590)	
Mean_Length	735.98 (486.84)	

### Sequence Annotation

We utilized several complementary approaches to annotate the assembled sequences. The unigenes were annotated by aligning with the deposited ones in diverse protein databases including National Center for Biotechnology Information (NCBI) non-redundant protein (Nr) database, NCBI non-redundant nucleotide sequence (Nt) database, UniProt/Swiss-Prot, Kyoto Encyclopedia of Genes and Genomes (KEGG), Cluster of Orthologous Groups of proteins (COG) and UniProt/TrEMBL. The best one was selected from the matches with an E-value of less than 10^−5^. The overall functional annotation was depicted in Table 3. First, a sequence similarity search was conducted against the NCBI Nr and Nt database, and Swiss-Prot protein database using the Basic Local Alignment Search Tool (BLAST) algorithm specifying E-values of less than 10^−5^. The analysis indicated that of the 68,229 unigenes, 46,087 (67.5%) had significant matches in the Nr database and 52,660 (77%) had significant matches in the Nt database while 28,165 (41.2%) unigenes had similarity to proteins in the Swiss-Prot database. Altogether, 54,884 (78.9%) unigenes were successfully annotated in the Nr, Nt, Swiss-Prot, KEGG, COG and TrEMBL databases listed in Table S1. Gui et al. (2010) produced 1.2 Mb of tetraploid Moso bamboo sequences from 13 bacterial artificial chromosome (BAC) clones, with 46% of 112 non-TE-related protein-coding genes predicted to be protein-encoding genes and displaying high similarity to genes of other plants deposited in the NCBI Genebank [27]. The significance of the BLAST comparison depends in part on the length of the query sequence. Short reads obtained from sequencing would rarely be matched to known genes [28]. The low percentage (21.1%) of unmapped unigenes that can be assigned a putative function might be mainly due to the short sequence reads generated by the sequencing technology and the relatively short sequences of the resulting unigenes, most of which probably lack the conserved functional domains [12]. Another possible reason is that some of these unigenes might be non-coding RNAs. The insufficient sequences of bamboo in public databases also influence the annotation efficiency [12]. Gui et al. (2010) showed that although both rice and sorghum exhibit high genomic synteny with bamboo, the comparison of the two bamboo-rice-sorghum syntenic regions demonstrated that some Moso bamboo genes seemed to have been lost or moved to other genomic regions after the divergence of bamboo from other members [27]. Meanwhile, some Moso bamboo genes have no hits to the syntenic region or even other regions of rice and sorghum, suggesting they might be bamboo-specific genes [27]. Therefore, generation of large collection of bamboo unigenes and ESTs is of great necessity for the bamboo research. Above all, these results demonstrated the reliability of Illumina paired-end sequencing and *de novo* assembly. According to the reads per kilo bases per million reads (RPKM) values, 5,000 genes were chosen to be high-expressed unigenes, among which 4,918 unigenes were annotated by NCBI Nr protein database (Table S2).

**Table 3:** Functional annotation of the *D. latiflorus* Munro transcriptome.

Annotated databases	All sequences	≥300 bp	≥1000 bp
nr_Annotation	46,087	25,385	6,306
nt_Annotation	52,660	28,065	6,282
swissprot_Annotation	28,165	15,800	5,506
GO_Annotation	11,921	6,329	3,547
kegg_Annotation	45,649	25,222	6,304
COG_Annotation	10,147	5,560	2,959
Total	54,893	29,083	6,343

**Figure 2:**
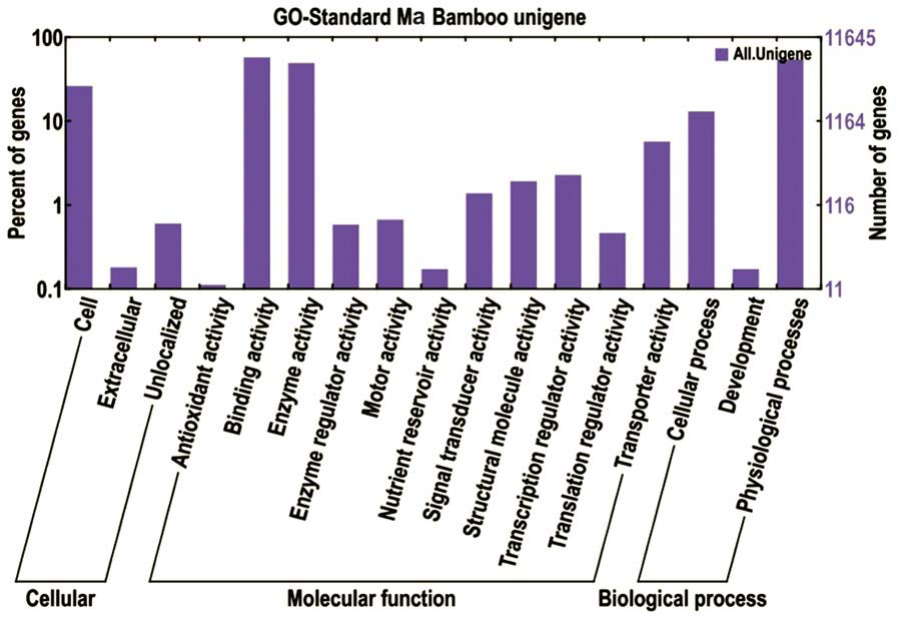
Functional annotation of assembled sequences based on gene ontology (GO) categorization. GO analysis was performed at the level 2 for three main categories (cellular component, molecular function and biological process).

Based on Nr annotation, Gene Ontology (GO) [29] analysis was carried out, which provides a dynamic, controlled vocabulary and hierarchical relationships for the representation of information on molecular function, cellular component and biological process, allowing a coherent annotation of gene products. There were 46,087 unigenes annotated in Nr, among which 11,921 unigenes were assigned with one or more GO terms, with 34.8% for biological processes, 50.6% for molecular functions, and 14.6% for cellular components (Fig 2). For biological processes, genes involved in physiological processes (GO: 0008152) and cellular processes (GO: 0009987) were highly represented. For molecular functions, binding activity (GO: 0005488) were the most represented GO term, followed by enzyme activity (GO: 0003824). Regarding cellular components, the most represented category was cells (GO: 0005623) (Fig 2).

**Figure 3:**
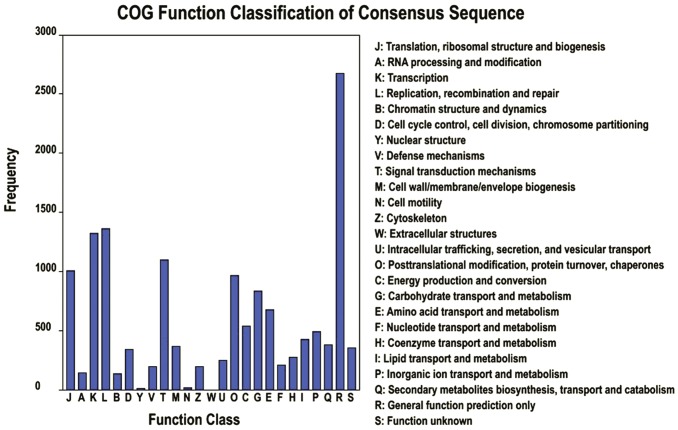
Clusters of orthologous groups (COG) classification. In total, 10,147 of the 68,229 sequences with Nr hits were grouped into 25 COG classifications.

In addition, all unigenes were subjected to a search against the COG database for functional prediction and classification. Overall, 10,147 of the 68,229 sequences showing a hit with the Nr database could be assigned to COG classifications (Fig 3). COG-annotated putative proteins were functionally classified into at least 25 protein families involved in cellular structure, biochemistry metabolism, molecular processing, signal transduction and so on (Fig 3). The cluster for general function prediction (2,673; 26.34%) represented the largest group, followed by replication, recombination and repair (1,359; 13.39%), transcription (1,319; 13%), signal transduction mechanisms (1,096, 10.8%), translation, ribosomal structure and biogenesis (1,004; 9.89%), posttranslational modification, protein turnover and chaperones (964; 9.5%), carbohydrate transport and metabolism (831, 8.19%), amino acid transport and metabolism (673; 6.63%), energy production and conversion (538; 5.3%), and whereas only a few unigenes were assigned to nuclear structure and extracellular structure (18 and 5 unigenes, respectively). In addition, 368 unigenes were assigned to cell wall/membrane/envelope biogenesis and 248 unigenes were assigned to intracellular trafficking, secretion and vesicular transport (Fig 3).

To further demonstrate the usefulness of Ma bamboo unigenes generated in the present study, we identified biochemical pathways represented by the unigene collection. Annotations of Ma bamboo unigenes were fed into the KEGG Pathway Tools, which is an alternative approach to categorize genes functions with the emphasis on biochemical pathways. This process predicted a total of 292 pathways represented by a total of 45,649 unigenes. Summary of the sequences involved in these pathways was included in Table S3. These predicted pathways represented the majority of plant biochemical pathways for compound biosynthesis, degradation, utilization, and assimilation, and pathways involved in the processes of detoxification and generation of precursor metabolites and energy. Enzymes catalyzing almost all steps in several major plant metabolic pathways including the Calvin cycle, glycolysis, gluconeogenesis, the pentose phosphate pathway, and several important secondary metabolite biosynthesis pathways including carotenoid biosynthesis and flavonoid and anthocyanin biosynthesis, could be represented by unigenes derived from the Ma bamboo dataset. Moreover, genes involved in several signaling pathway including the p53, mammalian target of rapamycin (mTOR), vascular endothelial growth factor (VEGF) and mitogen-activated protein kinase (MAPK) signaling pathway, were also found in the unigene collection.

### Comparative Analysis with Moso Bamboo and Other Grasses

To take a snapshot on the relationship between Moso bamboo and Ma bamboo in terms of orthology, or to identify proteins or pathways that might be unique to one of the two species, we did a new BLAST operation between the two datasets. A search for nucleotide sequence similarity with a relatively high stringency (E-value <1e-10 in BLASTn) showed that 1.6% or 870 of 54,884 unigenes had a significant match to Moso bamboo 10,608 putative FL-cDNAs. These unigenes were subjected to a search against the COG database for functional prediction and classification and there were 268 unigenes which could be assigned to COG classifications. The largest group was the cluster for general function prediction (59; 22%), followed by translation, ribosomal structure and biogenesis (55; 20.5%), and posttranslational modification, protein turnover and chaperones (40; 15%). The rest of these highly matched unigenes were predicted to play roles in energy production and conversion, and the transport and metabolism of carbohydrates, amino acids, nucleotides and lipids. Also, we subjected these highly matched unigenes to other databases including the NCBI Nr, NCBI Nt, SwissProt, and GO seqdb databases. The detailed results are listed in Table S4. It is noteworthy that a large number of unigenes had no hits to the Moso FL-cDNAs. This may be explained by the fact that the Moso cDNA database is incomplete in the sense that it was constructed only from shoots, leaves, and roots of germinating seeds. Nonetheless many of the unmatched Ma bamboo unigenes may be indeed unique. The functions of these unigenes remain to be further characterized.

**Figure 4:**
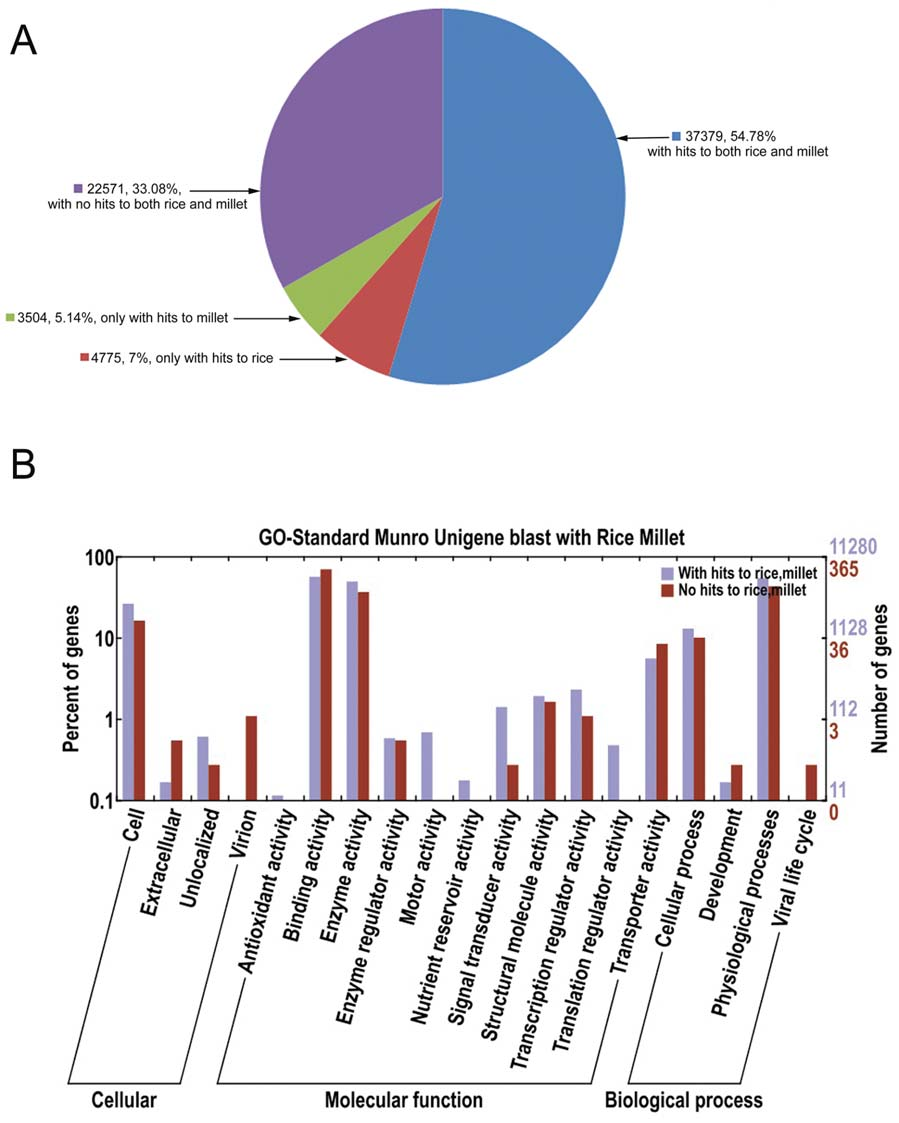
Ma bamboo unigene similarity comparison with rice and millet and functional classification by GO analysis. (A) Similarity search of Ma bamboo sequence against rice and millet. (B) Functional classification of Ma bamboo unigenes with and without homologs with rice and millet.

Given the wealth of rice and millet genome data, we examined at first the proportion of Ma bamboo unigenes that matched rice and millet databases at the nucleotide sequence level by a sequence similarity search. The search for nucleotide sequence similarity with a relatively high stringency (E-value <1e-10 in BLASTn) showed that 42,154 (61.78%) Ma bamboo unigenes had similarity hits to rice transcripts and 40,883 (59.92%) unigenes had similarity hits to millet transcripts. Among these aligned sequences, 37,379 unigenes had similarities with both rice and millet, while 4,775 unigenes only had similarity hits to rice and 3,504 unigenes only had similarity hits to millet (Fig 4A). A total of 22,571 (30.08%) bamboo unigenes did not match any of rice and millet sequences which were presumably Ma bamboo unique (Fig 4A). The detailed results are listed in Table S5.

Based on the above similarity search, we then conducted GO analysis to compare the functional classification between two groups of Ma bamboo unigenes, one including shared homologs with rice and millet and the other presumably being unique to Ma bamboo (Fig 4B). The detailed results were listed in Table S5. In all, among 37,379 shared homologs, there were 10,740 unigenes which were assigned with one or more GO terms. The GO analysis showed that for biological processes, genes involved in physiological processes (GO: 0008152) and cellular processes (GO: 0009987) were highly represented. For molecular functions, binding activity (GO: 0005488) were the most represented GO term, followed by enzyme activity (GO: 0003824). Regarding cellular components, the most represented category was cells (GO: 0005623) (Fig 4B). Only 423 of 22,571 unigenes predicted to be unique to Ma bamboo were annotated by GO analysis displaying a similar trend to the annotated shared homologs. The low annotation percent is probably due to the relatively small fraction of high-quality sequence-finished bamboo genes deposited and annotated in public databases, especially compared with rice and Arabidopsis. Bamboo is famous for its fast growth rate and high woodiness. Cui et al (2012) identified 213 spots differentially expressed in culm development by MALDI-TOF/TOF MS which were involved in many physiological and metabolic processes including carbohydrate metabolism, cell division, cell expansion, protein synthesis, amino acid metabolism and redox homeostasis [30]. Therefore we postulate that differences in the expression profile and the function allocation of the Ma bamboo unigenes with sequence similarity hits to rice and millet concurrently contribute to the divergence of bamboo from other grasses. Also, many of the predicted genes that are unique to Ma bamboo, and which encode proteins that are predominantly associated with binding activities and catalytic functions or that are involved in physiological processes, are likely to have defined bamboo as the plant species it is today. Although we cannot yet be certain about which genes precisely define bamboo as a species, we are convinced that the large bulk of predicted unigenes unique to Ma bamboo represent a valuable resource to explore Ma bamboo gene diversity and to allow for comparative genomic studies among grasses.

### Functional Genes Involved in Lignin Biosynthesis

As is well known, the lignin content of bamboo is higher than most herbaceous plants [31], which may be the result of differences in the number or level of expression of key enzymes involved in lignin biosynthesis. We identified 105 unigenes from the 54,884 unigenes encoding eight key enzymes involved in lignin biosynthesis (KEGG PATH: ko00940, http://www.genome.jp/kegg/) (Table S6). For each enzyme, we compared the number of putatively Ma bamboo unigenes in this study with the number of Moso bamboo FL-cDNAs identified from the Moso cDNA database and rice genes identified from the genome sequences (Table 4). Peng et al (2010) predicted 26 transcripts encoding 4-coumarate-CoA ligase (4CL) and 23 transcripts encoding laccase from the rice genome database [6]. Our results show that these two genes are also the most abundant ones involved in Ma bamboo lignin synthesis. However, the numbers of transcripts encoding for these two genes in Ma bamboo were higher than those in rice. There were 34 transcripts found for laccase and 35 transcripts coding for 4-coumarate-CoA ligase (4CL), which would partially contribute to the increased bamboo lignin content in comparison to rice. Among the 10,608 FL-cDNAs in Moso bamboo, the numbers of FL-cDNAs encoding 4CL and laccase were two and five respectively [6]. The significant difference between Ma bamboo and Moso bamboo is not surprising because the 10,608 Moso FL-cDNAs actually represent only one third to one fourth of the estimated total of Moso bamboo genes. Also, the Moso FL-cDNA libraries were constructed from shoots, leaves and roots from germinating seeds which were not the most representative tissues for high lignin content, whereas our materials used for the transcriptome sequencing covered as many tissues as possible including culms of different developing periods. As previously reported, laccase had been found to display high expression in lignifying tissues [32]. Therefore, the spatial distributions of genes related to lignin biosynthesis also influence the results. Of course, the differences among different species also count in the analysis. It is interesting that we could not detect unigenes coding for 5-hydroxyconiferyl aldehyde O-methyltransferase (AldOMT), which means that other genes encoding alternative methyltransferases, substituting for AldOMT activity, may exist in Ma bamboo. This needs to be further characterized. The above results indicate that lignin biosynthesis in Ma bamboo may follow yet unknown routes or pathways and that lignin synthesis in Ma bamboo displays unique features. However, as lignin biosynthesis is a complex process involving numerous factors, our analysis by transcriptome sequencing only elucidates part of the picture and hence it is difficult to draw precise conclusions. Clearly, additional studies deploying accurate molecular and proteomic analysis procedures are required to validate and further build on our predictions.

**Table 4:** Number of bamboo FL-cDNAs and number of genes found in the rice genome that encode nine key enzymes in the lignin biosynthesis pathway.

Enzymes	Ma bamboo	Moso bamboo^a^	Rice^a^
4-coumarate-CoA ligase (4CL)	35	2	26
Caffeoyl caffeoyl-CoA O-methyltransferase (CCoAOMT)	4	9	10
Cinnamoyl-CoA reductase (CCR)	10	7	18
Caffeic acid O-methyltransferase (COMT)	7	2	10
Cinnamate-4-hydroxylase (C4H)	4	1	4
Cinnamoyl alcohol dehydrogenase (CAD)	2	6	21
Laccase	34	5	23
5-hydroxyconiferyl aldehyde O-methyltransferase (AldOMT)	0	1	7
3-deoxy-D-arabino-heptulosonate 7-phosphate synthase (DAHPS)	7	2	8

a The results were cited from Peng et al (2010).

### Functional Genes Involved in Growth and Development

For many agricultural plants like bamboo, economic traits like growth and development are of particular interest to researchers. The sequence and annotation information from BLAST, GO and KEGG annotations all provided valuable gene sources for the study of molecular basis that underline these economic traits of Ma bamboo. Among them, genes encoding different groups of growth factors and their receptors involved in cell growth were identified, such as epidermal growth factor domains and receptors, transforming growth factors and receptors, hepatocyte growth factors and receptors and fibroblast growth factor and receptors.

It has been reported that many transcription factor families play vital roles in plant growth, development and immunity [33]–[37]. From BLASTn analysis, we identified sets of unigenes that have putative functions as transcription factors including those belonging to the zinc finger protein family, F-box family, WD repeat-containing protein family, Myb family, WRKY family, MADS-box family, GATA family, etc. These putative transcription factors likely play specific and diverse roles in regulating gene expression levels endowing bamboo with unique features.

Unlike other plants, bamboo flowering is an elusive physiological phenomena, because it is unpredictable, long-periodic and uncontrollable. We have identified genes belonging to zinc finger protein family, WD repeat-containing protein family and MADS-box family which are thought to be correlated with bamboo flowering [38]. Also, some other genes involved in flowering such as those coding for polycomb group protein [39], YABBY protein [38], phytochrome [40] and histone deacetylase [41], were identified in our Illumina dataset.

As a plant which has gained reputation as a major resource of non-wood fiber, genes related to molecular mechanism of its fiber development are yet to be explored. Some fiber related genes of bamboo [42], such as those encoding a kinase-like protein involved in fiber initiation, a heat shock protein HSP82 involved in fiber elongation, or an eukaryotic initiation factor 4A involved in fiber maturation, were identified in our dataset. Further studies are required to identify the genes associated with fiber development in particular those that contribute to the outstanding fiber characteristics of bamboo.

Moreover, genes encoding plant hormones could be identified in our dataset. As is well known, plant hormones determine the formation of flowers, stems, leaves, the shedding of leaves, and the development and ripening of fruit. There are 425 unigenes involved in the auxin pathway such as auxin-responsive proteins IAA and auxin response factors. We also identified 263 unigenes for the ethylene pathway and 92 unigenes related to the gibberellin pathway.

Overall, functional analysis of our Illumina dataset identified candidate genes potentially involved in growth, development, plant signal pathways and regulatory networks.

### SSR Discovery

SSRs are highly informative and widely used for genetics, evolution and breeding studies. It has been reported that approximately 3–7% of expressed genes contain putative SSR motifs, mainly within the un-translated regions of the mRNA [43]. SSRs within gene sequences may have different putative functions, for example, SSR variations in 5′-untranslated regions (UTRs) could regulate gene expression by affecting transcription and translation; SSR expansions in the 3′-UTRs cause transcription slippage and produce expanded mRNA; intronic SSRs can affect gene transcription, mRNA splicing, or export to cytoplasm; SSRs within genes should be subjected to stronger selective pressure than other genomic regions [44]. To explore SSR profiles in the unigenes of Ma bamboo, the 6,375 unigene sequences were submitted to an online service to search for SSRs [15]. In total, 621 SSRs were obtained from 824 unigenes (12.8%) with 121 unigene sequences containing more than one SSR, among which tri-nucleotide repeat motif was the most abundant, accounting for 56.03%, followed by di-nucleotide repeat motif (33.33%), tetra-nucleotide (2.41%), penta-nucleotide (1.12%), and hexa-nucleotide (0.48%) repeat units (Table 5). The relative percentage of unigenes containing SSRs was much smaller than that of Moso bamboo (24%), which might be attributed to sequencing sampling or species difference [6].

**Table 5:** Summary of simple sequence repeat (SSR) types in the *D. latiflorus* Munro transcriptome.

Repeat motif			Number^a^			Percentage (%)^b^	
**Di-nucleotide**							
AC/CA/GT/TG			36				
AG/GA/CT/TC			152				
AT/TA			13				
CG/GC			6				
**Total**			**207**		**33.33%**		
**Tri-nucleotide**							
AAC/AAG			4				
GAA/AGA/ACA/CTT/TTC/TCT/TTG			21				
TAA/ATT/TTA/ATA			6				
ACC/CAC/CCA/GGT/GTG/TGG			35				
ACG/GAC/CGA/CGT/GTC/TCG			21				
TAC/TAG/GTA/TCA/ACT			10				
AGC/CAG/GCA/TGC/CTG/GCT			69				
AGG/GGA/GAG/TCC/CTC/CCT			92				
ATC/CAT/GAT/ATG/TGA			25				
CCG/CGG/CGC/GCC/GCG/GGC			61				
**Total**			**348**			**56.03%**	
**Tetra-nucleotide**							
ATGC/ATGG/AAAT			4				
TGGA			1				
GAAA/GCAT/GTAG/GAGT/GCCG/GCAC			6				
CTCC/CAAC			4				
**Total**			**15**			**2.41%**	
**Penta-nucleotide**							
ACTGG/ATTGT			2				
CCCTG/CTTCC/CTGTG			3				
GAGAG/GATGG			2				
**Total**			**7**			**1.12%**	
**Hexa-nucleotide**							
CCACGG/TCAGGC/TTTTCT			3				
**Total**			**3**			**0.48%**	
**Compound SSR**			**41**			**6.60%**	

a Number of the total SSRs detected in unigenes

b The relative percentage of SSRs with different repeat motifs among the total SSRs

The AG/GA/CT/TC motifs accounted for approximately half of the total number of di-nucleotide SSRs, similar to that of *Huperzia serrata* Thunb [45]. Among the di-nucleotide repeat motifs, CT repeats were the most common, which is different from that of *H. serrate* or *Arabidopsis* in which AG repeats were the most frequent. This may be due to the introduction of additional repeats during chromosome replication [46]. It was reported that (CT)_n_ may function as an enhancer due to that fact that the same motif (TCTCTCTCT) was found in a 60-nt region downstream of the transcription start site of CaMV 35S RNA, which can enhance gene translation in plant protoplasts [47]. Furthermore, as complementary sequences to (CT)n, (GA)n serves as regulatory elements that contain a series of overlapped GAG motifs (AGAGAGa) involved in light regulation [48], [49]. When compared with the frequency of di- or tri-nucleotide motif of SSRs among the unigenes of Ma bamboo, the results were coincidence with those of *Arabidopsis*, rice and Moso bamboo, in which the type and distribution of tri-nucleotide SSRs were also the most abundant [6]. The most common motif for tri-nucleotide repeats of SSRs were CTC/CCT/TCC/GCT/GCA/GGA (38% of tri-nucleotides) and CGG/CGC/GGC (10.6% of tri-nucleotides), which are similar to those of Moso bamboo and rice. This phenomenon is perhaps correlated with the higher G+C content of grasses and may have allowed more frequent insertion/deletion of certain nucleotides, without causing frame shift mutations [50].

SSRs were developed as powerful molecular markers for comparative genetic mapping and genotyping since they are ubiquitous in transcriptomes, typically locus-specific and co-dominant, multi-allelic, highly polymorphic, and transportable among species within genera [51], [52]. EST databases have been a rich source of SSRs for genotyping in numerous species of flowering plants [53]. The unigenes obtained from Ma bamboo have provided a good resource for SSR mining and applications in research and molecular marker-assistant breeding.

### Conclusions

This work presents the first *de novo* transcriptome sequencing analysis of mixed RNA from Ma bamboo flowers, seeds and different tissues (root, leaf, shoot, stem) using the Illumina platform. 2.2 Gbp of data were generated and assembled into 68,229 unigenes. A large number of candidate genes potentially involved in growth, development, flowering and plant hormone pathways were identified, and are worthy of further investigation. Ma bamboo unigenes related to lignin biosynthesis were characterized and their sequences compared to the sequence databases of rice, millet, and Moso bamboo. Orthologous sequences and unigenes unique to Ma bamboo were preliminary classified. A large number of SSRs were also identified and ready for marker development. To our knowledge, this is the first application of Illumina paired-end sequencing technology to investigate the whole transcriptome of Ma bamboo and moreover the assembly of the reads was conducted without a reference genome. The dataset will improve our understanding of the molecular mechanisms of fiber development, lignin biosynthesis, flowering, and other biochemical processes in Ma bamboo. This resource should lay an important foundation for future genetic or genomic studies on bamboo species and will help to close a critical gap existing in grass comparative genomics and consequently allow the more efficient development of the grass system for evolutionary and functional studies of plant genes and genomes.

## Materials and Methods

### Ethics Statement

All necessary permits were obtained for the described field studies. The authority responsible for the bamboo garden is Nanjing Forestry Bureau which provides permissions to collect the samples for our scientific research.

### Plant Materials and RNA Extraction

*D. latiflorus* Munro was obtained from Nanjing bamboo garden, Fujian Province. Seeds, flowers and tissues including leaves, stem, shoots and root were dissected from the bamboos and immediately frozen and stored in liquid nitrogen until analysis. Total RNAs were extracted from these materials using the Norgan RNA Purification Kit (Norgan Biotek Corp., Ontario, Canada). The quality and quantity of total RNA was analyzed using an UltrasecTM 2100 pro UV/Visible Spectrophotometer (Amersham Biosciences, Uppsala, Sweden) and gel electrophoresis. Equal quantities of high-quality RNA from each material were pooled for cDNA synthesis.

### mRNA-seq Library Construction for Illumina Sequencing

The mRNA-seq library was constructed following the manufacturer’s instructions of mRNA-Seq Sample Preparation Kit (Cat # RS-930-1001, Illumina Inc, San Diego, CA) (Illumina). In briefly, the poly-(A) mRNA was isolated from the total RNA samples with Magnetic Oligo (dT) Beads. To avoid priming bias, the mRNA was fragmented by the RNA fragmentation kit (Ambion, Austin, TX) before cDNA synthesis. The cleaved RNA fragments were transcribed into first-strand cDNA using reverse transcriptase (Invitrogen, Carlsbad, CA) (Invitrogen) and random hexamer-primers, followed by second-strand cDNA synthesis using DNA polymerase I (New England BioLabs, Ipswich, MA) (NEB) and RNaseH (Invitrogen). The double-stranded cDNA was end-repaired using T4 DNA polymerase (NEB), the Klenow fragment (NEB), and T4 polynucleotide kinase (NEB) followed by a single <A> base addition using Klenow 3′ to 5′ exo-polymerase (NEB) to prepare the DNA fragments for ligation to the adapters, which have a single ‘T’ base overhang at their 3′ end, then ligated with PE Adapter Oligo Mix supplied by mRNA-Seq Sample Preparation Kit (Illumina) using T4 DNA ligase (NEB) and incubated at room temperature for 15 minutes. The products of the ligation reaction were purified according to the instructions of the MinElute PCR Purification Kit (QIAGEN, Dusseldorf, Germany) (QIAGEN) and eluted in 10 µL of QIAGEN EB buffer. The eluted Adaptor-ligated fragments of the ligation reaction were separated by size on an agarose gel to select a size range of templates for downstream enrichment. The desired range of cDNA fragments (200±25 bp) were excised and retrieved using a Gel Extraction Kit (Axygen Biosciences, Central Avenue Union City, CA). PCR was performed to selectively enrich and amplify the cDNA fragments using Phusion Master Mix (NEB) with two primers, PCR Primer PE 1.0 and PCR Primer PE 2.0 supplied by mRNA-Seq Sample Preparation Kit (Illumina). These primers anneal to the ends of the PE adapters under the conditions used: 30 seconds at 98°C; 15 cycles of 10 seconds at 98°C, 30 seconds at 65°C, 30 seconds at 72°C; 5 minutes at 72°C; hold at 4°C. The amplified products were purified according to the instructions of QIAquick PCR Purification Kit (QIAGEN) and eluted in 30 µL of QIAGEN EB buffer. Libraries were prepared from a 150–200 bp size-selected fraction following adapter ligation and agarose gel separation. The quality control analysis on the sample library was performed to quantify the DNA concentration and validate the library. After validation with an Eppendorf Mastercycler ep realplex Real-Time PCR System, the mRNA-seq libraries were sequenced using a single end read protocol with 32 bp of data collected per run on the Illumina Genome Analyzer IIx sequencing platform. Data analysis and base calling were performed by the Illumina instrument software.

### Sequence Data Analysis and Assembly

The raw reads were cleaned by removing adapter sequences, low-quality sequences (reads with ambiguous bases ‘N’), and reads with more than 10% Q <20 bases. All sequences smaller than 60 bases were eliminated based on the assumption that small reads might represent sequencing artifacts [23]. The quality reads were assembled into unigenes with Trinity which recovers more full-length transcripts across a broad range of expression levels, with sensitivity similar to methods that rely on genome alignments [54]. The overlap settings used for this assembly were 31 bp and 80% similarity, with all other parameters set to their default values.

### Sequence Annotation

The optimal assembly results were chosen according to the assembly evaluation. Then the clustering analysis was performed to achieve a unigene database which comprised the potential alternative splicing transcripts. SSR analysis of the unigenes which were longer than 1 kb was performed using the SSRIT software [15].

The assembled sequences were compared against the NCBI Nr and Nt database (Last updated on March 1st, 2011) and Swiss-Prot database using BlASTn (version 2.2.14) with an E-value of 10^−5^. Gene names were assigned to each assembled sequence based on the best BLAST hit (highest score). To increase computational speed, such search was limited to the first 10 significant hits for each query. Open reading frames (ORFs) were predicted using the “getorf” program of EMBOSS software package [55], with the longest ORF extracted for each unigene. We quantified transcript levels in reads per kilobase of exon model per million mapped reads (RPKM) [56]. The RPKM measure of read density reflects the molar concentration of a transcript in the starting sample by normalizing for RNA length and for the total read number in the measurement. Genes with high expression levels were screened and listed.

To annotate the assembled sequences with GO terms describing biological processes, molecular functions and cellular components, the Swiss-Prot BLAST results were imported into Blast2GO [57], [58], a software package that retrieves GO terms, allowing gene functions to be determined and compared. These GO terms are assigned to query sequences, producing a broad overview of groups of genes catalogued in the transcriptome for each of three ontology vocabularies, biological processes, molecular functions and cellular components. The obtained annotation was enriched and refined using ANNEX [59]. The data presented herein represent a GO analysis at level 2, illustrating general functional categories.

The unigenes sequences were also aligned to the COG database to predict and classify functions. KEGG pathways were assigned to the assembled sequences using the online KEGG Automatic Annotation Server (KAAS), http://www.genome.jp/kegg/kaas/. The bi-directional best hit (BBH) method was used to obtain KEGG Orthology (KO) assignment [60]. The output of KEGG analysis includes KO assignments and KEGG pathways that are populated with the KO assignments.

Moreover, we conducted comparative analyses against Moso bamboo FL-cDNAs (http://www.ncgr.ac.cn/MBCD/) with a relatively high stringency (E-value <1e-10 in BLASTn). The Ma bamboo unigenes with significant matches were applied to GO analysis for functional classification. We also compared Ma bamboo unigenes with currently available genome sequences of rice (IRGSP version 4.0: http://rgp.dna.affrc.go.jp/IRGSP/) and millet (http://foxtailmillet.genomics.org.cn/page/species/download.jsp) with BLASTn (E-value <1e-10).

### EST-SSR Detection

The 68,229 unigenes of Ma bamboo obtained in this study were also subjected to the detection of SSRs using the online program: Simple Sequence Repeat Identification Tool (SSRIT, http://www.gramene.org/db/markers/ssrtool) [15], [61]. The parameters were adjusted for identification of perfect di-, tri-, tetra-, penta-, and hexa-nucleotide motifs with a minimum of 6, 5, 4, 4, and 4 repeats, respectively. The report of this search included the total number of sequences containing SSRs among the submitted unigenes, sequence ID, SSR motifs, number of repeats (di-, tri-, tetra-, penta-, and hexanucleotide repeat units), repeat length, SSR starts, and SSR ends [61]. Mononucleotide repeats were ignored since distinguishing genuine mononucleotide repeats from polyadenylation products and single nucleotide stretch errors generated by sequencing was difficult.

## Supporting Information

Table S1 Sequences with significant BLASTn matches against Nr, Swiss-Prot database and other databases.
(XLS)

Table S2 4,918 of 5,000 highly expressed Sequences were annotated with significant BLASTn matches against Nr database.
(XLS)

Table S3 KEGG biochemical mappings for *Dendrocalamus latiflorus* Munro.
(XLSX)

Table S4 Putative homologs of Ma bamboo identified in Moso bamboo cDNA dataset and the annotation by NCBI Nr, Nt, Swiss-Prot and GO seqdb databases.
(XLS)

Table S5 Unigenes of Ma bamboo with hits to rice and millet and GO functions of unigenes of Ma bamboo with and without hits to rice and millet.
(RAR)

Table S6 Unigenes of Ma bamboo encoding eight key enzymes in the lignin biosynthesis pathway.
(XLSX)
